# Guidelines for reporting an fMRI study

**DOI:** 10.1016/j.neuroimage.2007.11.048

**Published:** 2008-04-01

**Authors:** Russell A. Poldrack, Paul C. Fletcher, Richard N. Henson, Keith J. Worsley, Matthew Brett, Thomas E. Nichols

**Affiliations:** a Department of Psychology, Department of Psychiatry and Biobehavioral Sciences, and Brain Research Institute, University of California Los Angeles, Los Angeles, CA 90095, USA; b Brain Mapping Unit, Department of Psychiatry, University of Cambridge, Cambridge, UK; c MRC Cognition and Brain Sciences Unit, Cambridge, UK; d Department of Mathematics and Statistics, McGill University, Montreal, Québec, Canada H3A 2K6; e GlaxoSmithKline Clinical Imaging Centre, London, UK

## Abstract

In this editorial, we outline a set of guidelines for the reporting of methods and results in functional magnetic resonance imaging studies and provide a checklist to assist authors in preparing manuscripts that meet these guidelines.

Students in the sciences are often taught that one goal of a scientific paper should be to allow other researchers to replicate their study. However, as scientific research becomes more complex, it is increasingly difficult to report all of the details necessary to allow exact replication. This lack of methodological details can also hinder understanding and assessment of reported results by reviewers and readers. In addition, precise specification of relevant methodological details is crucial to ensure that large-scale databases contain the metadata necessary for effective data mining and meta-analysis. Because neuroimaging is such a multidisciplinary science, papers may be written and read by physicists, physiologists, psychologists and statisticians, just to name a few possibilities. This poses a unique challenge since it is important to give sufficient detail that any of these readers will be satisfied that they understood what was done in the study.

Our goal in the present editorial is to present some straightforward guidelines aimed at making fMRI papers more complete in their description of methodological details and results. We describe and outline a set of guidelines for what details should be specified in any fMRI paper. Rather than specifying how a study should be done, we instead focus on what needs to be reported, regardless of how the study is done. In Appendix A, we present a more explicit checklist, which authors can use to ensure that their papers report all of the necessary information outlined in the guidelines. We realize that any guidelines must be responsive to changes in research methods over time and to differences in opinion regarding what should be reported. We are anxious too that this editorial should not be seen as an exercise in dogma or governance but rather as a starting point for encouraging debate aimed towards a widely accepted and flexible set of guidelines. With this in mind, a Wiki-based web site has been established at http://www.fmrimethods.org/. At this site, researchers can debate and collaboratively edit the guidelines, which should ensure that they reflect current standards in the field rather than the opinion of a select set of researchers. The web site also presents an example of a complete methods section that follows the proposed guidelines to serve as a guide for implementing the guidelines.

## Describe both who the subjects were and who they were not

It is standard to provide basic demographic information about the participants in a study, but additional information is necessary to provide a full description. First, any inclusion and exclusion criteria beyond those implied in the demographics should be described (e.g., “Subjects reported no history of psychiatric or neurological disorders, and no current use of any psychoactive medications”). If the subject sample was recruited in a targeted manner, then the nature of the sampling strategy should be noted. In addition, it is important to note how many subjects were excluded from the study after the data were collected, and specifically why they were excluded.

## Describe both what the subjects were asked to do and what they actually did

When describing a psychological task used in fMRI, you should aim to provide sufficient detail that another experimenter could implement the task in a way that is functionally equivalent to the reported procedure; although this is often difficult even with papers from the experimental psychology literature, it is a worthy goal nonetheless. Many details matter; the psychophysicist may be concerned with the visual angle and luminance of the stimuli, whereas the economist may focus on details of how payment was determined. Writers must use their judgment to decide which details are important for a specific study, but a general rule is that it is better to include too much detail than too little.

## Know what “Talairach space” is and what it is not

The term “Talairach space” has become a potent source of confusion in neuroimaging, and researchers need to be careful when using it. A brain or atlas is in Talairach space if the anterior and posterior commisures are on the same horizontal line (the AC–PC line) and the midline plane contains this line. The fact that a brain is in Talairach space does not imply any particular brain shape or size, and in particular, does not mean that such a brain matches a particular template—such as the original Talairach atlas or the MNI/ICBM template. In fact, there are substantial differences between the original atlas described by Talairach and the MNI305 template that is most commonly used today (Brett et al., 2002; Devlin and Poldrack, 2007). In addition, even when the same template is used, different software packages can result in significant differences in the localization of specific structures in 3D space (Van Essen and Dierker, 2007). Therefore, reporting coordinates as being in ‘Talairach space’ without more details is too generic to be useful. It is critical that you specify the atlas or template that you have matched to. You should also give the specific details of the spatial normalization method, including the type of transformation used, and what kind of image is being transformed. Imaging papers often label activations according to Brodmann areas; if you do this, be clear how the label was identified (nearest coordinate in Talairach daemon, cytoarchitectonic definitions from the SPM Anatomy toolbox, etc). This issue is of particular importance for databasing efforts, which require the accurate mapping of data into a common space across datasets produced using different methods.

## Specify how regions of interest were determined

Regions of interest (ROIs) may be used either to extract estimates of evoked signals or to limit corrections for multiple tests to a subset of all voxels (Poldrack, 2007). In either case, it is essential that the paper describes how the ROIs were determined. It is particularly important that ROIs used for multiple test correction (often called “small volume correction”) are determined independently of the specific test on which the correction is performed, either using an orthogonal contrast or an independent scan. If ROIs are determined anatomically, then the rules for anatomical demarcation should be specified explicitly (e.g., “the inferior frontal gyrus pars triangularis was defined as the region bounded dorsally by the inferior frontal sulcus, ventrally by the lateral fissure, posteriorly by the ascending ramus of the lateral fissure and anteriorly by the horizontal ramus of the lateral fissure, as described by Petrides and Pandya, 2004”). If the ROIs are functionally defined, then the specific contrast used to define them should be specified. We recommend that researchers provide ROI definitions in some appropriate format in the Supplementary material of the paper.

## Provide enough detail to reproduce the analysis

While very powerful, fMRI analysis packages can produce results that are easily misinterpreted or, more problematic, have advanced features that can be misused. To ensure that you and your reader exactly understand the model, it is essential that the approach be described in detail. Although most fMRI studies now report analyses using the general linear model (GLM), there remain substantial differences in how these models are specified and estimated. To a great degree these differences can be captured by knowing which software package was used to perform the analysis, but there can be substantial variability within packages depending upon which options are chosen. Whenever possible, provide a rationale for the user-specified parameters of the software. Some of the important details that may vary even within a package include how the error covariance structure is modeled (e.g., temporal autocorrelation in fMRI timeseries, or correlation induced by repeated measures across subjects). Even within the framework of GLM-based analyses, there are many different approaches to building models. For task-related regressors, it is important to be clear about how the task was modeled (e.g., for a blocked design, was the model based on a boxcar or a series of impulses for each trial within a block?) and how the BOLD impulse response was modeled (e.g., a single or dual-gamma canonical hemodynamic response, or a finite impulse response basis set?). If other regressors such as motion parameters or behavioral covariates are included these should also be described, as should any measures to orthogonalize these regressors. One increasingly common way to present GLM-based design matrices is as an image, which is available from most statistical packages. It is also important to describe the how group effects, as opposed to those in individual subjects, were analyzed and, finally, what precise statistical tests formed the basis for inferences reported. The comparisons that have been performed should be clearly specified in terms of which regressors were included in the contrast and be related to the hypotheses that these comparisons are meant to test.

The majority of published studies today use methods that are part of established software packages and have been described in methodological publications. However, it is not uncommon for a paper to present results using a method that has not been previously described in a methodological publication. In this case, it is critical that the method be described in algorithmic detail so that it can be reproduced by others. We encourage researchers to do this by making their code available with their publication as the most complete description of the procedure. It may also be useful to attach an appendix that describes the method, either mathematically or with pseudocode.

The best test of reproducibility is allowing others to directly reproduce the analysis on your own data. We strongly encourage researchers to make their raw data publicly available with their publication, e.g., via a central database or local web site.

## Report statistical tests to support all claims

Any empirical claim that is reported should be supported by a specific statistical test. While this may seem obvious, it is a principle that is often violated in the neuroimaging literature. Most commonly, this occurs when an author observes that activation is present in one comparison but absent in another comparison and concludes on this basis that there is a difference in the two effects; Henson (2005) referred to this as the “imager's fallacy” due to its prevalence in the literature. However, presence versus absence of a significant effect across two comparisons (e.g., groups) does not demonstrate a significant difference between the two; demonstrating that the two effects are different requires a direct statistical comparison of the effects. Likewise, claims about differences in activation across hemispheres or regions must be supported by a significant interaction. It is critical to note that identification of a significant regional response does not imply that this region is uniquely or more strongly involved in the process of interest compared to other regions, merely that, while the null hypothesis has been refuted in this region, it has not been so refuted elsewhere. Authors should try to avoid implying that their activated region is the only region involved in the task. If they do wish to directly assert that one or more other regions was not active, this assertion should be accompanied by effect sizes, confidence intervals, or Bayesian posterior probabilities for the effect.

## Always describe and account for the multiple testing problem

fMRI provides an embarrassment of riches due to the high dimensionality of the data, but this comes with the cost of a high risk of type I error due to the very large number of concurrent statistical tests. Hence it is essential that authors specify the magnitude of the multiple testing problem and how this issue is dealt with. The severity of the problem is described by the number of voxels tested and smoothness of the data (the estimated smoothness, not applied smoothness, if reported by the software). Examples of specific approaches to multiple testing include voxel- or cluster-wise control of family-wise error, voxel-wise control of the false discovery rate, or formal heuristics which have been shown (in peer reviewed publication) to control false positives in some objective manner. Be clear about the inferences that can be drawn from your approach. For example, if you have used an uncorrected threshold then state clearly that you have unquantified control of family-wise error. Corrected or both corrected and uncorrected inferences should be reported and clearly labeled according to the type of correction. When cluster-based inference is used, this should be clearly noted and both the threshold used to create the clusters and the threshold for cluster size should be reported. Finally, while thresholds must account for the multiplicity of tests, we do encourage authors to make available unthresholded statistic and effect size images in order to display the whole range of effects in the data, including those that do not reach significance. These maps also make it easier to compare effect sizes across studies and increase the options for future meta-analyses.

## Figures and tables should stand on their own

The effective presentation of fMRI results often involves presentation of figures with thresholded color-coded statistical maps or presentation of tables listing locations of significant activation. For figures, important details include the nature of the statistical map, the intensity and cluster size threshold used to create the image, the identity of the underlying anatomical image, and any additional operations that have been performed to the map (such as masking out particular regions). It is helpful to put these details in the caption. It is best to present statistical maps at the same threshold used in the results section, but if different thresholds are used for the figure and results text, then this must be clearly specified. For multi-contrast experiments, plots of effect size for each contrast (e.g., condition) in a given region of interest can be helpful, though it is important to indicate how the ROI was identified. Likewise, tables should include information about the nature of the statistical map and thresholding operations. Minimum data to be included in a table should include location of activation in stereotactic space (e.g., that of the maximum for voxel-level inference), statistics regarding the activation cluster (including maximum statistic value and size of the cluster), and anatomical labels. The means by which the anatomical labels are derived (e.g., an atlas or automated labeling method) should be clearly specified. We also recommend that tables or figures include some form of effect size measure (e.g., mean percent signal change and standard deviation) in order to allow future meta-analyses.

The question often arises as to how data should best be presented. There are many acceptable forms for presentation of fMRI results, from bar graphs to maximum intensity projections (‘glass brains’) to full color cortical surface renderings, and each has its rightful place. Our general recommendation is that the nature of the data presentation should follow from the hypotheses that are being tested. Thus, if hypotheses are being tested at the group level, it likely makes most sense to present group-averaged maps, whereas a study that is testing hypotheses about individual differences should present some representation of the data that makes these differences clear (e.g., scatterplots or boxplots).

## Quality control measures should be documented

There is a broad range of quality control measures that are applied in fMRI data acquisition and analysis, with no common set of measures or methods across laboratories. We encourage both the use and the detailed documentation of quality control measures in order to provide reviewers and readers with the best possible ability to estimate the presence of potential problems with the data or analysis. One particular measure that we recommend is the presentation (either in supplementary materials or in a downloadable online format) of the voxel mask used in the group data analysis, which demonstrates which voxels were included in the analysis. In our experience, examination of the mask can provide a quick way to determine the presence of a number of problems with the data. In recommending the presentation of data for the purposes of quality control, we follow the example of other fields, such as human genetics (Chanock et al., 2007) and gene microarrays (Shi et al., 2006).

## Conclusion

Instituting a more consistent and coherent policy for the reporting of fMRI methods should ensure that reviewers and readers of publications have the greatest possible ability to understand and potentially reconstruct the methods employed in the study. Furthermore, we believe that the generally accessible web page may help promote a broadly collaborative approach to defining and refining these guidelines and, in so doing, may promote their wider acceptance. We realize that this could result in published papers that are longer, but the costs of such lengthening should be outweighed by a more effective literature, and the ability to publish online supplementary materials in many journals also facilitates the presentation of more extensive methodological details without lengthening the main text.

Many other areas of bioscience are currently undergoing similar debates regarding minimal information standards for methodological reporting, such as the MIAME guidelines for microarray research (Brazma et al., 2001) and the CONSORT guidelines for clinical trials (Begg et al., 1996), and we hope that the fMRI community will join us in working towards a community standard for fMRI methods reporting. In some areas (e.g., clinical trials), checklists like the one in our Appendix A are required to be completed for submission of papers. We hope neuroimaging journals will consider this requirement.

## Figures and Tables

**Table:** 

All designs	
Number of blocks, trials or experimental units per session and/or subject	
Length of each trial and interval between trials	
If variable interval, report the mean and range of ISIs and how they were distributed	
Blocked designs	
Length of blocks	
Event-related designs	
Was the design optimized for efficiency, and if so, how?	
Mixed designs	
Report correlation between block and event regressors	

**Table:** 

Instructions	
What were subjects asked to do?	
Stimuli	
What were the stimuli and how many were there?	
Did specific stimuli repeat across trials?	

**Table:** 

If the experiment has multiple conditions, what are the specific planned comparisons, or is an omnibus ANOVA used?	

**Table:** 

Number of subjects	
Age (mean and range)	
Handedness	
Number of males/female	
Additional inclusion/exclusion criteria, if any (including specific sampling strategies that limit inclusion to a specific group, such as laboratory members)	
If any subjects were scanned but then rejected from analysis after data collection, state how many and reasons for rejection	
For group comparisons, what variables (if any) were equated across groups?	

**Table:** 

State which IRB approved the protocol	

**Table:** 

How was behavioral performance measured (e.g., response time, accuracy)?	

**Table:** 

MRI system:	
Manufacturer, field strength (in Tesla), model name	
MRI acquisition:	
Number of experimental sessions and volumes acquired per session	
Pulse sequence type (gradient/spin echo, EPI/spiral)	
If used, parallel imaging parameters (e.g., method [SENSE/GRAPPA] and acceleration factor)	
Field of view, matrix size, slice thickness, interslice skip	
Acquisition orientation (axial, sagittal, coronal, oblique; if axials co-planar with AC–PC, the volume coverage in terms of *Z* in mm)	
Whole brain? if not, state area of acquisition (preferably with a figure)	
Order of acquisition of slices (sequential or interleaved)	
TE/TR/flip angle	

**Table:** 

For each piece of software used, give the version number (or, if no version number is available, date of last application of updates)	
If any subjects required different processing operations or settings in the analysis, those differences should be specified explicitly	

**Table:** 

Specify order of preprocessing operations	
Describe any data quality control measures	
Unwarping of B0 distortions	
Slice timing correction	
Reference slice and type of interpolation used (e.g., “Slice timing correction to the first slice as performed, using SPM5's Fourier phase shift interpolation”)	
Motion correction	
Reference scan, image similarity metric, type of interpolation used, degrees-of-freedom (if not rigid body) and, ideally, optimization method, e.g., “Head motion corrected with FSL's MCFLIRT by maximizing the correlation ratio between each timepoint and the middle volume, using linear interpolation.”	
Motion susceptibility correction used	

**Table:** 

Intersubject registration method used	
Illustration of the voxels present in all subjects (“mask image”) can be helpful, particularly for restricted fields of view (to illustrate overlap of slices across all subjects). Better still would be an indication of average BOLD sensitivity within each voxel in the mask	
Transformation model and optimization	
Transformation model (linear/affine, nonlinear), type of any non-linear transformations (polynomial, discrete cosine basis), number of parameters (e.g., 12 parameter affine, 3 × 2 × 3 DCT basis), regularization, image-similarity metric, and interpolation method	
Object image information (image used to determine transformation to atlas)	
Anatomical MRI? Image properties (see above)	
Co-planar with functional acquisition?	
Functional acquisition co-registered to anatomical? if so, how?	
Segmented gray image?	
Functional image (single or mean)	
Atlas/target information	
Brain image template space, name, modality and resolution	
e.g., “FSL's MNI Avg152, T1 2 × 2 × 2 mm”; “SPM2's MNI gray matter template 2 × 2 × 2 mm”)	
Coordinate space	
Typically MNI, Talairach, or MNI converted to Talairach	
If MNI converted to Talairach, what method? e.g., Brett's mni2tal?	
How were anatomical locations (e.g., gyral anatomy, Brodmann areas) determined? (e.g., paper atlas, Talairach Daemon, manual inspection of individuals' anatomy, etc.)	

**Table:** 

Size and type of smoothing kernel (provide justification for size; e.g., for a group study, “12 mm FHWM Gaussian smoothing applied to ameliorate differences in intersubject localization”; for single subject fMRI “6 mm FWHM Gaussian smoothing used to reduce noise”)	

**Table:** 

For novel methods that are not described in detail in a separate paper, provide explicit description and validation of method either in the text or as an appendix	

**Table:** 

Statistical model and estimation method	
Multiple regression is most common statistical model	
Estimation methods are typically ordinary least squares (OLS), OLS with adjustment for autocorrelation (i.e., variance correction and use of effective degrees-of-freedom), or generalized least squares (i.e., OLS after whitening)	
Block/epoch-based or event-related model	
Hemodynamic response function (HRF)	
Assumed HRF model (e.g., SPM's canonical difference of gammas HRF; FSL's canonical gamma HRF), HRF basis (list basis set) or estimated HRF (supply methods for estimating HRF)?	
Additional regressors used (e.g., temporal derivatives, motion, behavioral covariates)	
Any orthogonalization of regressors	
Drift modeling/high-pass filtering (e.g., “DCT with cut off of *X* seconds”; “Gaussian-weighted running line smoother, cut-off 100 seconds”, or “cubic polynomial”)	
Autocorrelation model type (e.g., AR(1), AR(1) + WN, or arbitrary autocorrelation function), and whether global or local.	
(e.g., for SPM2/SPM5, ‘Approximate AR(1) autocorrelation model estimated at omnibus *F*-significant voxels (*P* < 0.001), used globally over the whole brain’; for FSL, ‘Autocorrelation function estimated locally at each voxel, tapered and regularized in space.’).	
Contrast construction	
Exactly what terms are subtracted from what? Define these in terms of task or stimulus conditions (e.g., using abstract names such as AUDSTIM, VISSTIM) instead of underlying psychological concepts	

**Table:** 

Statistical model and estimation method, inference type (mixed/random effects or fixed), e.g., “Mixed effects inference with one sample *t*-test on summary statistic” (SPM2/SPM5), e.g., “Mixed effects inference with Bayesian 2-level model with fast approximation to posterior probability of activation.” (FSL)	
If fixed effects inference used, justify	
If more than 2-levels, describe the levels and assumptions of the model (e.g., are variances assumed equal between groups)	
Repeated measures?	
If multiple measurements per subject, list method to account for within subject correlation, exact assumptions made about correlation/variance	
e.g., SPM: “Within-subject correlation estimated at *F*-significant voxels (*P* <0.001), then used globally over whole brain”; or, if variances for each measure are allowed to vary, “Within-subject correlation and relative variance estimated…”	

**Table:** 

Type of search region for analysis, and the volume in voxels or CC	
If not whole brain, state how region was determined; method for constructing region should be independent of present statistic image	
If threshold used for inference and threshold used for visualization in figures is different, clearly state so and list each	
Explicitly state if inferences are corrected for multiple comparisons, and if so, what method and over what region	
If correction is limited to a small volume, the method for selecting the region should be stated explicitly	
If no formal multiple comparisons method is used, the inference must be explicitly labeled “uncorrected”	
Voxel-wise significance? Corrected for Family-wise error (FWE) or false discovery rate (FDR)?	
If FWE found by random field theory list the smoothness in mm FWHM and the RESEL count	
If FWE found by simulation (e.g., AFNI AlphaSim), provide details of parameters for simulation	
If not a standard method, specify the method for finding significance (e.g., “Custom in-lab software was used to construct statistic maps and thresholded at FDR< 0.05 (Benjamini and Hochberg, 1995)”	
Cluster-wise significance	
State cluster-defining threshold (e.g., *P* = 0.001)	
State the corrected cluster significance level	
(e.g., “Statistic images were assessed for cluster-wise significance using a cluster-defining threshold of *P* = 0.001; the 0.05 FWE-corrected critical cluster size was 103”)	
If significance determined with random field theory, then smoothness and RESEL count must be supplied	
Correction for multiple planned comparisons within each voxel?	
False negative discussion	
Any discussion of failure to reject the null hypothesis (e.g., lack of activation in a particular region) should be accompanied by SNR or effect size of the actually observed effect (allows reader to infer power to estimate an effect)	

**Table:** 

How were ROIs defined	
(e.g., functional versus anatomical localizer)?	
How was signal extracted within ROI?	
(e.g., average parameter estimates, FIR deconvolution?)	
If percent signal change reported, how was scaling factor determined (e.g., height of block regressor or height of isolated event regressor)? Is change relative to voxel-mean, or whole-brain mean?	

**Table:** 

What statistical map is the figure/table based upon (e.g., *Z*, *t*, *p*)?	
Thresholds used to create the image or figure (intensity and cluster extent, where appropriate)	
**Figures**	
What is the underlying anatomical image (e.g., average anatomy, template image)?	
Any additional operations (e.g., masking out parts of the image)?	
**Tables**	
Locations in stereotactic space (with the space described specifically)	
Statistics for each cluster (including maximum and cluster extent)	
Source of anatomical labels (e.g., atlas, automated labeling method)	

## Appendix A Guidelines for experimental presentation

***Experimental design***

**Design specification**

**Task specification**

**Planned comparisons**

***Human subjects***

**Details on subject sample**

**Ethics approval**

**Behavioral performance**

***Data acquisition***

**Image properties—as acquired**

***Data preprocessing***

**Pre-processing: general**

**Intersubject registration**

**Smoothing**

***Statistical modeling***

**General issues**

**Intrasubject fMRI modeling info**

**Group modeling info**

***Statistical inference***

**Inference on statistic image (thresholding)**

**ROI analysis**
